# Partitioning of CH_4_ and CO_2_ Production Originating from Rice Straw, Soil and Root Organic Carbon in Rice Microcosms

**DOI:** 10.1371/journal.pone.0049073

**Published:** 2012-11-05

**Authors:** Quan Yuan, Judith Pump, Ralf Conrad

**Affiliations:** Max-Planck-Institute for Terrestrial Microbiology, Marburg, Germany; University of California Irvine, United States of America

## Abstract

Flooded rice fields are an important source of the greenhouse gas CH_4_. Possible carbon sources for CH_4_ and CO_2_ production in rice fields are soil organic matter (SOM), root organic carbon (ROC) and rice straw (RS), but partitioning of the flux between the different carbon sources is difficult. We conducted greenhouse experiments using soil microcosms planted with rice. The soil was amended with and without ^13^C-labeled RS, using two ^13^C-labeled RS treatments with equal RS (5 g kg^−1^ soil) but different δ^13^C of RS. This procedure allowed to determine the carbon flux from each of the three sources (SOM, ROC, RS) by determining the δ^13^C of CH_4_ and CO_2_ in the different incubations and from the δ^13^C of RS. Partitioning of carbon flux indicated that the contribution of ROC to CH_4_ production was 41% at tillering stage, increased with rice growth and was about 60% from the booting stage onwards. The contribution of ROC to CO_2_ was 43% at tillering stage, increased to around 70% at booting stage and stayed relatively constant afterwards. The contribution of RS was determined to be in a range of 12–24% for CH_4_ production and 11–31% for CO_2_ production; while the contribution of SOM was calculated to be 23–35% for CH_4_ production and 13–26% for CO_2_ production. The results indicate that ROC was the major source of CH_4_ though RS application greatly enhanced production and emission of CH_4_ in rice field soil. Our results also suggest that data of CH_4_ dissolved in rice field could be used as a proxy for the produced CH_4_ after tillering stage.

## Introduction

Flooded rice fields are an important source of the greenhouse gas CH_4_ [1], [2]. Methane and CO_2_ are end products of anoxic degradation of organic matter in rice field soil [3]. The organic matter is mainly derived from three sources [4]: (1) soil organic matter (SOM), (2) root organic carbon (ROC) including root exudates and sloughed-off dead root, and (3) dead plant organic matter, such as rice straw (RS), which is often applied in large amounts (up to 12 t ha^−1^ annually) to maintain soil fertility [5]–[7]. Methane production is partitioned mainly between these three types of organic matter. Knowledge of partitioning is important for improving process-based modeling of CH_4_ emission from rice fields [8], [9], which is the basis for predicting methane flux and assessing the impact of agricultural management and global change.

Quantification of carbon partitioning can in principle be achieved by pulse-labeling of rice plant with ^13^CO_2_ or ^14^CO_4_ [10]–[12]. Recently, free-air CO_2_ enrichment (FACE) using ^13^C-depleted CO_2_ was used for determining the contribution of ROC to production of CO_2_ and CH_4_ in rice field soil [13]. However, pulse-labeling only assesses the immediate contribution of root exudates, while the contribution of sloughed-off dead root cells cannot be fully accounted for [13]–[16]. Since FACE experiments apply elevated CO_2_ concentrations, photoassimilation of CO_2_ may be enhanced and thus increase the contribution of plants and soil organic matter to carbon flux [17]–[19]. Furthermore, most studies of carbon flux partitioning in rice fields have been done without application of straw, so that full partitioning of the origin of carbon flux into SOM, ROC and RS was not possible [4]. However, application of RS should be taken into account, since RS may not only be used as substrate for CH_4_ production, but might also enhance CH_4_ production from other carbon sources [20], [21].

The partitioning of the CH_4_ production from different sources of organic carbon (SOM, ROC, RS) can be achieved, if these have different isotopic signatures. However, a major difficulty during partitioning the sources of CH_4_ is caused by the carbon isotopic fractionation during the conversion of organic matter to CH_4_, which is typically 10–70‰ [22]. Nevertheless, the relative contribution of acetoclastic versus hydrogenotrophic methanogenesis to CH_4_ production has been determined successfully in environments such as rice field soil [23] and lake sediments [24], after the isotopic fractionation factors in both methanogenic pathways were determined. The δ^13^C values of CH_4_ from the two pathways are substantially different, since the isotopic fractionation factors of the two pathways are largely different [22], . Analogously, it is possible to partition the sources of CH_4_ if the δ^13^C of CH_4_ derived from each carbon source in the rice field soil is known. Normally, the CH_4_ derived from SOM, ROC and RS has similar δ^13^C values, since all the organic matter has eventually been derived from rice plant material [23], [26]. However, this problem may be solved by cultivation of rice in soil amended with ^13^C-labeled RS.

The aim of this study was to determine the partitioning of the carbon flux involved in methanogenic degradation of carbon sources by determining the δ^13^C of CH_4_ derived from ROC. We therefore prepared rice microcosms with two treatments of ^13^C-labeled RS, both having the same amount of RS (5 g kg^−1^ soil, equals about 5 t ha^−1^) but different content of ^13^C. We determined the produced CH_4_ and CO_2_ by collecting soil cores and incubating samples anoxically [27].

## Materials and Methods

### Planted and unplanted rice microcosms

Soil samples were provided by the Italian Rice Research Institute in Vercelli. Soil was taken from a drained paddy field in spring 2009 and was air dried and stored at room temperature. The soil was sieved (<2 mm) prior to use. The characteristics of the soil have been described previously [28]. Planting pots (upper diameter  = 19 cm; lower diameter  = 14 cm; height  = 16 cm) were filled with 2 kg dry soil and turned into a slurry with demineralized water.

For planted rice microcosms, in total 48 pots were prepared, 16 pots for the unamended control, and 16 pots each for RS treatment I and RS treatment II. Fertilizer solution (50 ml of a solution containing per liter: 10 g urea, 7.6 g KH_2_PO_4_) was added to each pot as basal fertilizer. For both RS treatments, 10 g powder of RS was added to each pot and mixed thoroughly into the soil slurry. The δ^13^C values of RS added in treatment I and II were 213.0‰ and 474.7‰, respectively. These δ^13^C values were obtained by adding desired amount of ^13^C-labeled (δ^13^C = 1859.9‰) and unlabeled (δ^13^C = −27.6‰) RS separately into each pot. The ^13^C-labeled RS was prepared by growing rice plants in the greenhouse until the late vegetative stage. The plants were covered with a 18-L acrylic chamber, 1% ^13^CO_2_ (final concentration; 99 atom%, Sigma, Germany) was added to the headspace, incubated for 5 days (12 h light, 25°C), and then harvested. The unlabeled RS was from rice plant grown in the same manner without feeding on ^13^CO_2_. These rice plants were dried and ground to powder. After 3 days of incubation in the greenhouse, all the pots were planted with one 12-day old rice seedling (*Oryza sativa* var. KORAL type japonica), and were flooded with demineralized water to give a water depth of 5 cm above the soil surface. The water depth was maintained throughout the experimental period. The rice microcosms were incubated in the greenhouse with a relative humidity of 70%, a 12-h photoperiod and a 28/22°C day/night temperature cycle. The day of transplantation was taken as day zero. On day 21, a second dose of 30 ml fertilizer solution was added to each microcosm. At each sampling time (day 41, 55, 70 and 90), 12 rice microcosms were sacrificed (4 replicates for control and for each treatment). For unplanted microcosms, the preparation was the same as for planted ones, but without rice plant in the pots. In total, 12 pots were prepared with 4 pots each for the unamended control, RS treatment I and RS treatment II.

### CH_4_ flux, soil pore water and plant parameters

Rates of CH_4_ emission was measured on day 41, 55, 70 and 90 of incubation in the greenhouse as described previously [27]. For flux measurements, planted rice microcosms were covered by flux chambers, and gas samples were taken every 30 min for 2 h. CH_4_ emission rates were determined from the slope of the linearly increasing CH_4_ mixing ratio and expressed in mmol CH_4_ m^−2^ h^−1^.

Samples for the determination of the isotopic signature (δ^13^CH_4_) of the emitted CH_4_ were taken in glass containers (100 ml). The first sample was taken directly after closure of the chambers, the second sample was taken at the end of the 2-h closure period. The isotopic signature of the emitted CH_4_ was calculated according to [27].

Pore water samples were collected into Venoject blood-collecting tubes (Terumo Europe N.V., Belgium) from the rhizosphere (3 cm depth) and bulk (9 cm depth) soil of rice microcosms using Rhizon pore water samplers (Rhizosphere Research Products, the Netherlands). After heavy shaking by hand, the headspace of the tubes was sampled using a pressure lock syringe and directly analyzed for CH_4_ and CO_2_ and δ^13^C. The CH_4_ and CO_2_ concentration in the soil pore water was calculated as described previously [27].

Plant height, tiller number and aboveground biomass were determined. For dry weight determination, samples were dried for 48 h at 60°C.

### CH_4_ and CO_2_ production

Production rates of CH_4_ and CO_2_ and respective δ^13^C values were determined by collecting soil core samples in rice microcosms on day 41, 55, 70 and 90 of incubation in the greenhouse [27]. After cutting off the rice plant, the surface water layer was removed. Soil cores were taken in each pot with stainless steel corer (Ø 22 mm, 210 mm in length). Two to three soil cores (about 100 g in total) were collected from each pot and transferred into a 250-ml bottle. The soil samples were turned into slurry using N_2_-gassed deionized sterile water so that the ratio of dry weight of soil to water was 1∶1. After flushing the samples with N_2_, the bottles were sealed with butyl rubber stoppers and, after shaking, flushed again with N_2_ to remove residual O_2_ and CH_4_. Incubation was performed statically at 25°C in the dark for 24 h. Headspace samples were taken every 12 h after shaking the bottles, and analyzed for concentration of CH_4_ and CO_2_ and their δ^13^C. The CH_4_ and CO_2_ production from planted soil microcosms was due to decomposition of SOM plus ROC (unamneded control) or of SOM, ROC plus RS (RS treatments). CH_4_ production rates were calculated by linear regression of the CH_4_ increase with incubation time, and expressed in nmol CH_4_ g_dw_^−1^ h^−1^ of soil. The CO_2_ production rates were determined analogously.

For unplanted soil microcosms, the methods for collection and incubation of soil core samples were similar, but these pots were not sacrificed, but at each sampling day (day 41, 55, 70 and 90), a 60-g soil core was taken from the pot. After removal of the soil core the residual soil in the pot was compacted, and water was added to maintain a water level of 5 cm depth. Using this procedure about 2.1% of the total amount of soil in the pot was collected during each sampling. The CH_4_ and CO_2_ production from unplanted soil microcosms was only due to decomposition of SOM (unamneded control) or of SOM plus RS (RS treatments).

### Analytical techniques

The gas samples were analyzed for CH_4_ and CO_2_ using a gas chromatograph (GC) equipped with flame ionization detector (FID) [29]. Stable isotopic analysis of gas samples (CH_4_ and CO_2_) from pore water and soil core incubation were performed directly using the GCC-IRMS, samples from flux measurements (low in CH_4_) were preconcentrated on a Precon (Finnigan, Bremen, Germany). The principal operation of the GCC-IRMS has been previously described [30], [31]. The isotope reference gas was CO_2_ (99.998% purity; Messer-Griessheim, Düsseldorf, Germany) calibrated with the working standard methyl stearate (Merck). The latter was intercalibrated at the Max-Planck-Institute for Biogeochemistry, Jena, Germany (courtesy of Dr. W.A. Brand) against NBS 22 and USGS 24, and reported in the delta notation vs. V-PDB: δ^13^C  = 10^3^ (*R*_sa_/*R*_st_ −1), with R  = ^13^C/^12^C of sample (sa) and standard (st), respectively. The precision of repeated analysis was ± 0.2‰, when 1.3 nmol CH_4_ were injected [23]. The determination of the stable isotopic signatures of dried plant and soil samples was carried out at the Institute for Soil Science and Forest Nutrition (IBW) at the University of Göttingen, Germany.

### Calculations

#### 1. Fraction of CH_4_ production from ROC (*f_ROC_*)

The fraction of CH_4_ derived from ROC (f_ROC_) can be determined from the following mass balance equation:
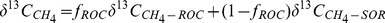
(1)where δ^13^C_CH4_  =  δ^13^C of CH_4_ produced (or dissolved) in the planted rice microcosms at each sampling time; δ^13^C_CH4-ROC_  =  δ^13^C of CH_4_ formed from ROC (determination see below); δ^13^C_CH4-SOR_  =  δ^13^C of CH_4_ formed from SOM plus RS, i.e. the CH_4_ produced (or dissolved) in the unplanted soil treated with RS. The equation can be transformed into the following two equations for RS-treatment I and II, respectively:



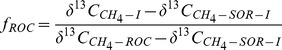
(2)

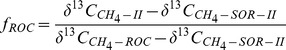
(3)


Since *f*_ROC_ and δ^13^C_CH4-ROC_ should be the same in treatment I and II, δ^13^C_CH4-ROC_ can be calculated by solving equations (2) and (3):
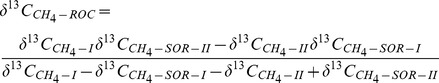
(4)


Then, *f*_ROC_ can be calculated from either equation (2) or (3).

#### 2. Fraction of CH_4_ production from RS carbon (*f_RS_*)

**Figure 1:**
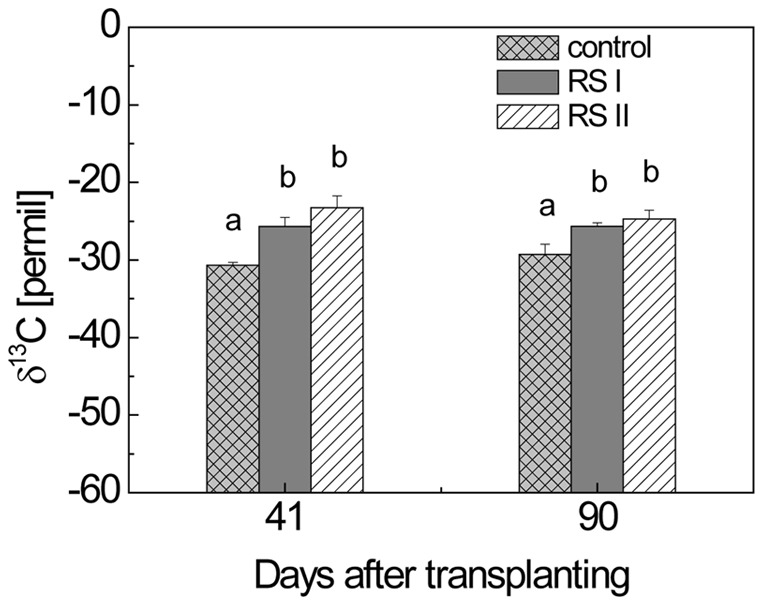
Values of δ^13^C of dried rice plants obtained from planted microcosms without (control) and with addition of ^13^C-labeled RS. RS I and RS II denote the two treatments, the δ^13^C of rice straw applied was 213.0‰ and 474.7‰ for RS I and RS II, respectively; means ± standard deviation (SD) (n = 3). The differences between the treatments over time were examined using Duncan *post hoc* test of a one-way ANOVA. Different letters on the top of bars indicate significant difference (*P*<0.05) between the data.

The δ^13^C values of the CH_4_ produced (or dissolved) in the two RS treatments are given by the following two mass balance equations:
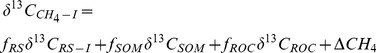
(5)

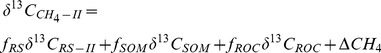
(6)with *f*_RS_, *f*_SOM_ and *f*_ROC_ denote fractions of CH_4_ produced from RS, SOM and ROC, respectively; δ^13^C_RS-I_ and δ^13^C_RS-II_ are δ^13^C of the rice straw carbon in treatment I (213.0‰) and II (474.7‰), respectively; δ^13^C_SOM_ and δ^13^C_ROC_ are δ^13^C of SOM (−25.8‰) and of the plant biomass (Fig. 1), respectively; ΔCH_4_ designates the overall isotopic fractionation factors involved in CH_4_ production from these organic matters, in case of dissolved CH_4_ also those involved in oxidation and transfer of CH_4_ from soil to the atmosphere.

Since the terms *f*_SOM_ δ^13^C_SOM_, *f*_ROC_ δ^13^C_ROC_ and ΔCH_4_ should be the same in treatment I and II, combination of equations (5) and (6) and solving for *f*_RS_ results in:
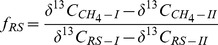
(7)of which the δ^13^C can be determined experimentally. Here, δ^13^C_CH4-I_ and δ^13^C_CH4-II_ were determined experimentally, and δ^13^C_RS-I_ and δ^13^C_RS-II_ were mixtures of labeled and unlabelled RS, of which the δ^13^C were determined experimentally (see above). Finally, the fraction of CH_4_ production from SOM (*f*_SOM_) can be calculated, since



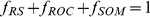
(8)Analogous equations are valid for the fractions of CO_2_ produced from ROC, SOM and RS in rice field soil.

### Statistical analysis

The significance of differences between treatments over time for various variables were determined by one-way analysis of variance (ANOVA) followed by multiple comparisons (Duncan *post hoc* test) using SPSS 13.0. To test the significance of the differences between contributions to produced and dissolved CH_4_ or CO_2_, two-tailed independent t-tests were applied using Microsoft Excel 2007.

## Results

### 1. Stable carbon signature of rice plants

The δ^13^C of rice plants in the control and RS treatments were almost constant with time (Fig. 1). Rice plants in the RS treatments were enriched in δ^13^C by about 5‰ compared with the control. The δ^13^C of rice plants was consistently higher in treatment II than in treatment I, but the difference was not significant.

### 2. Rates and δ^13^C of CH_4_ emitted from planted microcosms

**Figure 2:**
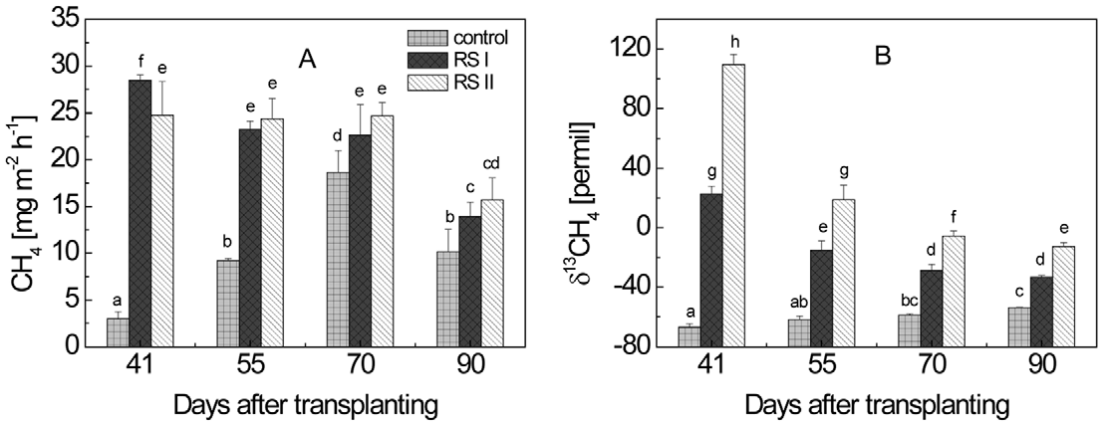
Seasonal change of (A) CH_4_ emission rates and (B) δ^13^C of CH_4_ emitted in planted microcosms with and without treatment with ^13^C-labeled RS; means ± SD (n  = 4). The differences between the treatments over time were examined using Duncan *post hoc* test of a one-way ANOVA. Different letters on the top of bars indicate significant difference (*P*<0.05) between the data.

In the rice microcosms without addition of RS, CH_4_ emission rates increased from the tillering stage (day 41) to the booting stage (day 55) and peaked at the flowering stage (day 70), then decreased again till the ripening stage (day 90) (Fig. 2A). Application of rice straw increased CH_4_ emission rates throughout the growth period, but particularly during tillering and booting stage (Fig. 2A). The δ^13^C of the emitted CH_4_ became gradually more negative during the cultivation period in all the treatments (Fig. 2B). The δ^13^C of CH_4_ was substantially higher in RS treatment II > RS treatment I > control, especially during the tillering stage (Fig. 2B).

### 3. Concentrations and δ^13^C values of CH_4_ and CO_2_ dissolved in pore water

**Figure 3:**
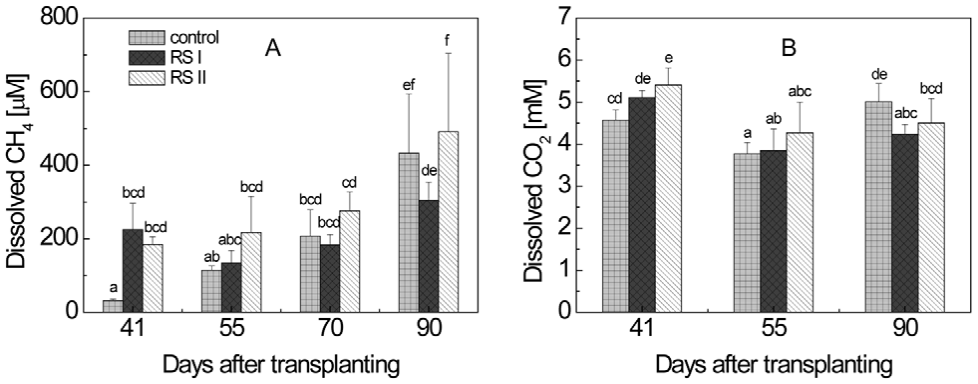
Temporal change of the concentrations of dissolved (A) CH_4_ and (B) CO_2_ in planted microcosms with and without addition of ^13^C-labeled RS; means ± SD (n = 4). The differences between the treatments over time were examined using Duncan *post hoc* test of a one-way ANOVA. Different letters on the top of bars indicate significant difference (*P*<0.05) between the data.

**Figure 4:**
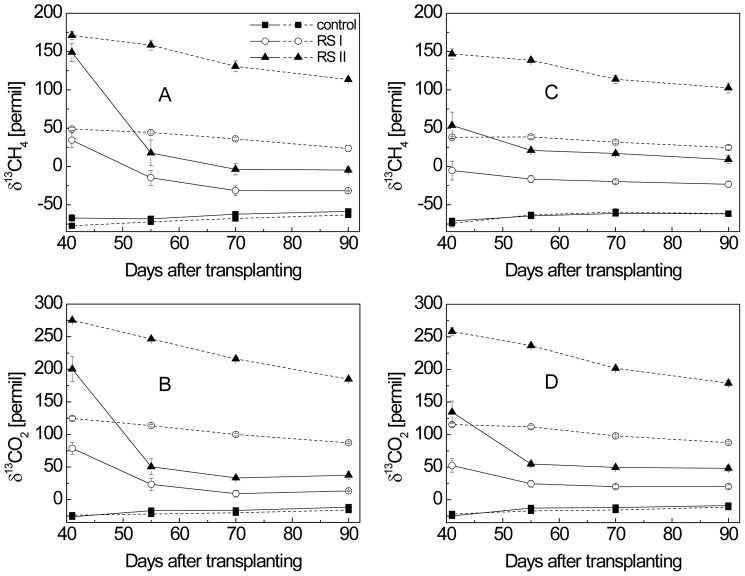
δ^13^C of (A) CH_4_ and (B) CO_2_ dissolved in microcosms with and without RS application; δ^13^C of (C) CH_4_ and (D) CO_2_ produced in microcosms with and without RS application. Solid line indicated planted microcosms, dashed lines unplanted microcosms; means ± SD (n = 4).

Concentrations and δ^13^C values of dissolved CH_4_ and CO_2_ were similar in the pore water sampled from 3 cm and 9 cm soil depth. Therefore, only the data from the 9-cm soil layer are shown (Fig. 3, 4A and B). In the planted microcosms, CH_4_ concentrations increased steadily from the beginning until the ripening stage. Application of rice straw resulted in elevated CH_4_ concentrations in the beginning but subsequently became similar to the control (Fig. 3A). The δ^13^C values of the CH_4_ dissolved in planted and unplanted microcosms were similar with each other in both RS treatments at tillering stage (Fig. 4A). However, while δ^13^C values decreased with time in the planted microcosms, they did not decrease much in the unplanted microcosms. The δ^13^C of the dissolved CH_4_ was consistently higher (less negative) in RS treatment II > RS treatment I > control for both planted and unplanted microcosms (Fig. 4A). The δ^13^C values of the dissolved CH_4_ in planted microcosms (Fig. 4A) were similar to those of the emitted CH_4_ (Fig. 2B).

In the planted microcosms, dissolved CO_2_ concentrations were between 4.0 and 5.5 mM independently of the treatment and the vegetation period (Fig. 3B). The δ^13^C of the dissolved CO_2_ exhibited a temporal pattern similar to that of CH_4_ and was again consistently higher (less negative) in RS treatment II > RS treatment I > control (Fig. 4B). However, δ^13^C of dissolved CO_2_ was in general higher (less negative) than that of CH_4_.

### 4. Rates and δ^13^C of CH_4_ and CO_2_ produced in planted and unplanted microcosms

**Figure 5:**
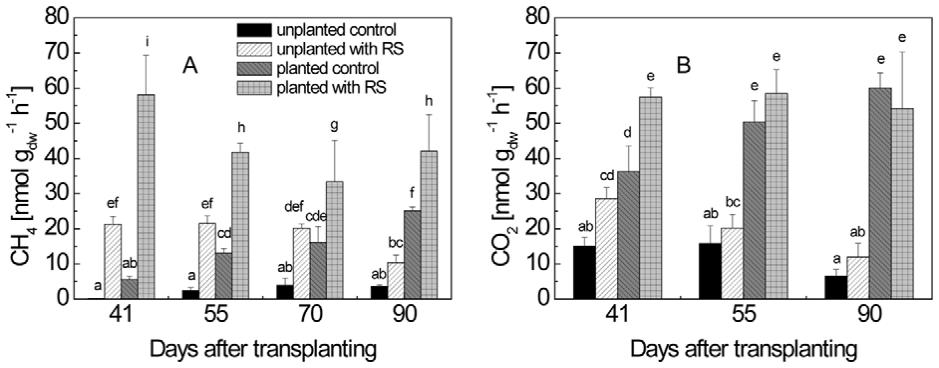
Production rates of (A) CH_4_ and (B) CO_2_ in planted and unplanted microcosms with and without RS application; means ± SD (n = 4). The differences between the treatments over time were examined using Duncan *post hoc* test of a one-way ANOVA. Different letters on the top of bars indicate significant difference (*P*<0.05) between the data.

At each time of sampling, soil cores were collected from microcosms with and without rice plants, in order to determine the rates and the δ^13^C of the CH_4_ and CO_2_ produced. Depending on the microcosm tested, CH_4_ and CO_2_ were produced from ROC (planted microcosms), SOM (all microcosms) and RS (RS-treated microcosms). In the planted control without RS treatment, CH_4_ production rates increased steadily during the vegetation period (Fig. 5A). However, treatment with RS resulted in further increase of CH_4_ production rates. In the unplanted microcosms, CH_4_ production rates were also enhanced by RS treatments but were lower than in the planted microcosms with RS treatment. The δ^13^C of produced CH_4_ was similar in the planted and unplanted control microcosms without RS (Fig. 4C). Treatment with RS resulted in increase of δ^13^C values of produced CH_4_, which was higher in treatment II than treatment I. However, the increase was less in the planted than in the unplanted microcosms (Fig. 4C).

The rates of CO_2_ production were constant over the vegetation period in the planted microcosms and were similar for the treatments with and without RS, but were at least twice as high in planted as in unplanted microcosms (Fig. 5B). The δ^13^C values of CO_2_ exhibited a similar pattern with respect to vegetation period and treatment as that of CH_4_, but the values were generally higher (Fig. 4D).

### 5. Partitioning CH_4_ and CO_2_ produced in rice microcosms

**Figure 6:**
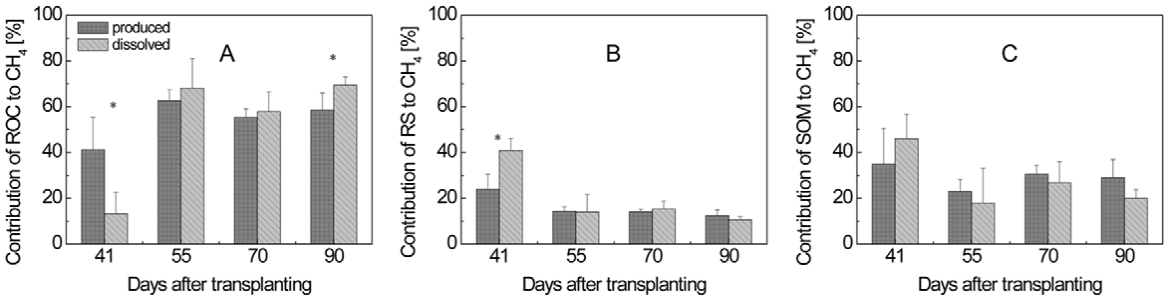
Percentage contribution of (A) ROC, (B) SOM and (C) RS to produced and dissolved CH_4_ in planted microcosms with RS treatment; means ± SD (n = 4). The differences between contributions to produced and dissolved CH_4_ were tested by two-tailed independent t-tests, indicated by ^*^ when *P*<0.05.

**Figure 7:**
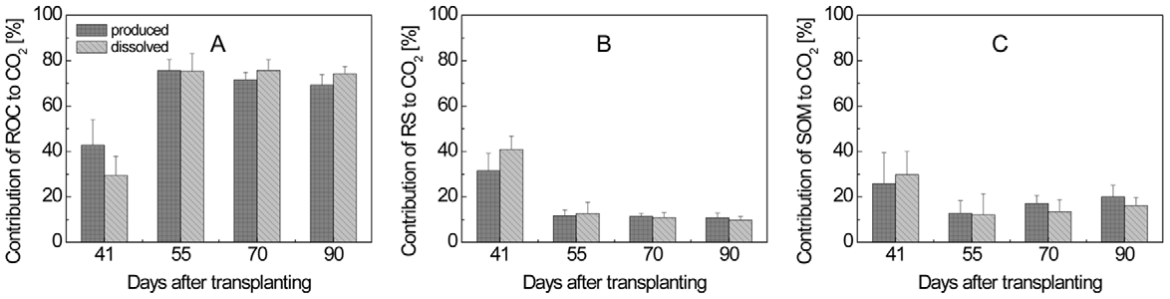
Percentage contribution of (A) ROC, (B) SOM and (C) RS to produced and dissolved CO_2_ in planted microcosms with RS treatment; means ± SD (n = 4). The differences between contributions to produced and dissolved CH_4_ were tested by two-tailed independent t-tests, indicated by ^*^ when *P*<0.05.

For calculation of *f*_ROC_, first of all the δ^13^C of the CH_4_ and CO_2_ produced from ROC had to be determined. The data, which were calculated using eq. (4), are shown in Table 1. The δ^13^C of CH_4_ produced from ROC was about −60‰ on average (range of −67 to −49‰) during the whole vegetation period, though fluctuations on individual sampling dates, at tillering stage in particular, were rather high (Table 1). The δ^13^C values of CO_2_ produced from ROC were about −31‰ at tillering stage and increased to around −11‰ to −4‰ subsequently (Table 1). Values of *f*_ROC_ were then calculated using eq. (2) and (3). Both equations gave similar values, but those obtained with eq. (2) showed higher standard deviations than those obtained with eq. (3). Only the latter values are shown in Fig. 6 and 7. ROC was found to make a major contribution (41–63%) to CH_4_ production over the entire vegetation period (Fig. 6A). For CO_2_ production, ROC had even a higher importance (43–76%) (Fig. 7A).

**Table 1:** δ^13^C values of CH_4_ and CO_2_ derived from ROC in planted rice microcosms with RS application.

Days after transplanting	41	55	70	90
δ^13^C_CH4-ROC_	−67.4±66.7	−49.4±14.2	−61.3±10.2	−57.2±17.4
δ^13^C_CO2-ROC_	−31.3±65.1	−3.6±14.6	−10.7±8.8	−9.7±10.6

The values were calculated using δ^13^C of CH_4_ and CO_2_ produced in rice field soil; means ± SD (n = 4).

The fractions of CH_4_ and CO_2_ produced from RS (*f*_RS_) were calculated using eq. (7). Values of δ^13^C were obtained from the CH_4_ (Fig. 4C) and CO_2_ (Fig. 4D) produced in soil samples from planted microcosms. Values of *f*_RS_ were determined to be in a range of 12–24% for CH_4_ production (Fig. 6B) and 11–31% for CO_2_ production (Fig. 7B).

Finally, *f*_SOM_ was calculated by difference to *f*_ROC_ and *f*_RS_, being in a range of 23–35% of CH_4_ (Fig. 6C) and 13–26% of CO_2_ production in soil from planted and straw-treated microcosms (Fig. 7C).

### 6. Partitioning CH_4_ and CO_2_ dissolved in rice microcosms

Similarly as for the production of CH_4_ and CO_2_ (see above), the gases dissolved in the rice microcosms were also used for determination of the partitioning of their origin from ROC, RS, and SOM using the equations described above. In this case, values of δ^13^C were from the CH_4_ and CO_2_ dissolved in pore water of planted and unplanted microcosms (Fig. 4A and B). The δ^13^C of CH_4_ derived from ROC was −30‰ at tillering stage when calculated with δ^13^C of CH_4_ in pore water (Table 2), substantially more positive than that calculated with δ^13^C of produced CH_4_ (Table 1). The resulting *f*_ROC_ for CH_4_ was only 13% (Fig. 6A). In contrast, the relative contribution of RS (*f*_RS_) to CH_4_ dissolved was significantly higher than that for CH_4_ produced at the tillering stage (Fig. 6B). However, the relative contributions of each carbon source to dissolved and produced CH_4_ were nearly the same at later season (Fig. 6). For CO_2_, the δ^13^C of CO_2_ derived from ROC was −49‰ at tillering stage, more negative than that calculated with δ^13^C of produced CO_2_ (−31‰), but there was no significant difference between the relative contributions of each carbon source to dissolved and produced CO_2_ (Fig. 7).

**Table 2:** δ^13^C values of CH_4_ and CO_2_ derived from ROC in planted rice microcosms with RS application.

Days after transplanting	41	55	70	90
δ^13^C_CH4-ROC_	−29.9±95.2	−38.7±25.4	−72.2±28	−51.0±7.6
δ^13^C_CO2-ROC_	−49.2±81.1	−3.8±22	−14.2±14.2	−8.5±6.1

The values were calculated using δ^13^C of CH_4_ and CO_2_ dissolved in pore water; means ± SD (n = 4).

## Discussion

### 1. Partitioning of methane production

Our study comprehensively determined the partitioning of CH_4_ and CO_2_ production in a rice ecosystem considering all three major carbon sources (i.e., ROC, RS, SOM). In planted and straw-treated rice microcosms, more than 60% of the CH_4_ was produced from root organic carbon, except on the first sampling date (tillering stage) when it was 41%. Thus, plant photosynthesis was the most important driver of CH_4_ production. The same was the case for CO_2_ production. The results are consistent with the observation that CH_4_ and CO_2_ production rates were at least twice as high in microcosms with than without rice plants (Fig. 5A and 5B). At the same time, the substantial lower δ^13^C of CH_4_ and CO_2_ produced in planted versus unplanted microcosms also indicated that ROC-derived CH_4_ and CO_2_ production diluted the CH_4_ and CO_2_ produced from labeled rice straw (Fig. 4C and 4D). Our results are consistent with two earlier experiments reporting 40–60% of the CH_4_ production being due to plant derived carbon. These experiments were based on pulse-labeling and FACE techniques [11], [13], which potentially influence carbon flux partitioning in a different way than our approach. For instance, pulse-labeling may only account for part of the plant-derived carbon flux and FACE treatment may stimulate carbon flux [13], [14]. Nevertheless, the determined relative contribution of plant derived carbon to production of CH_4_ and CO_2_ was rather similar despite the different approaches. Hence, the results that plant-derived carbon is the most important carbon source for CH_4_ production in flooded rice fields is a rather robust finding.

In contrast to ROC, straw contributed only about 20% to CH_4_ production. A similar low percentage has previously been found in Japanese rice soil microcosms after 50 days of incubation [4]. Immediately after application of the straw, however, its contribution to CH_4_ production and emission reached almost 100% [4]. This was likely also the case in our experiments. This conclusion is supported by the following observations: (1) On day 41, δ^13^C of the produced CH_4_ was <150‰ albeit the applied rice straw carbon had a δ^13^C of 474.7‰ (Fig. 4C). The difference is much more than theoretically possible from isotope discrimination during methanogenesis. Therefore, we have to assume that the CH_4_ produced immediately after straw application had a much higher δ^13^C as it was derived from straw to a large extent. (2) The analogous observation was made with the produced CO_2_ (Fig. 4D), although isotope discrimination is much smaller for production of CO_2_ than of CH_4_. (3) Still after day 40, δ^13^C of the produced CH_4_ and CO_2_ tended to decrease with vegetation time. Hence, we conclude that contribution of decomposition of straw to CH_4_ production was very high after straw application and then progressively decreased as the carbon compounds of the straw became increasingly less decomposable. Future studies should further refine the seasonal change in flux partitioning. This will help improving the predictions of CH_4_ emission rates from rice fields by process-based modeling.

### 2. Contribution of different carbon sources to the dissolved CH_4_ and CO_2_

Previous studies reported that δ^13^C values of pore water CH_4_ and emitted CH_4_ were relatively poor proxies for those of produced CH_4_ [32], [33]. This assessment is plausible, since in rice field soil pore water CH_4_ and emitted CH_4_ are not only affected by CH_4_ production, but also by CH_4_ oxidation [34]–[36] and CH_4_ transport [37]–[39], which all undergo carbon isotopic fractionation. Therefore, we primarily used the CH_4_ produced in soil samples for determining flux partitioning. However, we found that not only the data of the produced CH_4_ but also of the dissolved CH_4_ allowed determination of flux partitioning and resulted in similar values. Thus, more than 60% of the CH_4_ and CO_2_ dissolved in soil pore water were derived from root organic carbon after tillering stage, nearly the same as for produced CH_4_ and CO_2_ (Fig. 6 and 7).

At tillering stage, however, the relative contribution of ROC to the dissolved CH_4_ was significantly lower and that of RS significantly higher when compared to the contribution to the produced CH_4_. The difference was probably due to the gas transport limitation of rice plants at the early vegetative stage [32], [40]. The residence time of CH_4_ in pore water at tillering stage can amount to several days. Therefore, at day 41 the pore water was probably still highly enriched in ^13^CH_4_ which had been produced from RS at earlier time. This conclusion is consistent with the substantially higher δ^13^C values of the dissolved CH_4_ than those of the produced CH_4_ at day 41 (Fig 4A and 4C). As a result, the relative contribution of RS to dissolved CH_4_ was higher than to produced CH_4_ at day 41 and that of ROC was lower (Fig. 6B).

In contrast, at later growth season, the residence time of CH_4_ in pore water of planted soil was much shorter (several hours) [32], this was consistent with the rapid decrease of δ^13^C values of dissolved CH_4_ and CO_2_ after tillering stage. Furthermore, the δ^13^C values of dissolved and produced CH_4_ were similar with each other after the tillering stage (Fig. 4A and 4C). Therefore, the relative contributions of each carbon source to dissolved and produced CH_4_ were similar to each other (Fig. 6). This suggested that pore water CH_4_ could be used as a proxy for produced CH_4_ and could be suitable for partitioning the CH_4_ production after tillering stage.

### 3. Stable carbon isotope fractionation during CH_4_ production from ROC

The δ^13^C of the CH_4_ produced from ROC (δ^13^C_CH4-ROC_) were in a range of −67‰ to −49‰. These values are similar to δ^13^C_CH4_ values observed in rice field soil or in incubations of soil slurries [23], [33]. Theoretically the value of δ^13^C_CH4-ROC_ should be equal to the δ^13^C of ROC plus the overall isotopic enrichment factor (ε_ROC,CH4_) for the conversion of ROC to CH_4_. The δ^13^C_ROC_ should be similar to the δ^13^C of the rice plant biomass (Fig. 1). Using these values and the δ^13^C_CH4-ROC_, the overall enrichment factor ε_ROC,CH4_ was in a range of about −24‰ to −42‰. This is a rather large range, but has been observed before (about −20‰ to −75‰) during anaerobic decomposition of straw in paddy soil [41] or anoxic incubations of rice roots [42]. The overall enrichment factor ε_ROC,CH4_ is composed of (1) the enrichment factors involved in the conversion of ROC to the methanogenic substrates (i.e., acetate and H_2_/CO_2_) and (2) in the enrichment factors involved in the conversion of the methanogenic substrates to CH_4_. The latter enrichment factors are the larger ones, in particular those involved in the production of CH_4_ from H_2_/CO_2_ [23], [43]. Whereas acetoclastic methanogenesis has relatively moderate enrichment factors (−10‰ to −25‰), those of hydrogenotrophic methanogenesis are often very large (−25‰ to −90‰) [22]. Our data suggest that CH_4_ production from ROC is dominated by hydrogenotrophic methanogenesis, which is consistent with earlier observations studying CH_4_ production on rice roots [42], [44], [45].

The δ^13^C of the CO_2_ produced from root organic carbon was in a range of −31‰ to −4‰ (Table 1). The overall isotopic enrichment factors involved in CO_2_ production from organic matter were thus about −6‰ to +21‰. These enrichment factors are much smaller than those involved in CH_4_ production. Nevertheless, the range is similarly large, which may be due to carbon isotopic fractionation during CO_2_ consumption by hydrogenotrophic methanogenesis [23] and also during reactions between gaseous CO_2_ and bicarbonate/carbonate [46].

### 4. Practical considerations

Our study demonstrated the possibility to determine the partitioning of CH_4_ and CO_2_ flux from degradation of straw, soil organic matter, and plant root-derived carbon, by treating soil with ^13^C-labeled rice straw. The procedure is more practical than labeling of the rice plants with ^13^CO_2_ that requires cumbersome incubation techniques or expensive FACE treatment. For calculation of *f*_ROC_, it was important that the δ^13^C of the two RS applications were sufficiently different from each other, and in addition were sufficiently different from the δ^13^C of both ROC and SOM. This was achieved by two RS treatments using the same amount of RS but ^13^C-labeled to different extent. As a result, the δ^13^C of emitted CH_4_ (Fig. 2B), δ^13^C of dissolved and produced CH_4_ and CO_2_ (Fig. 4) were substantially higher than the control without RS, and of course they were always higher in treatment II than treatment I.

Calculation of *f*_RS_ was simply achieved by using the δ^13^C values of the applied RS and the CH_4_ derived from the two RS treatments (Eq. 7) assuming that ROC was not differently affected by the two RS treatments. This assumption was in agreement with the observation that the ^13^C values of the rice plants in the two RS treatments were not significantly different (Fig 1). Notably, these values were significantly higher than those in the control microcosms without RS, probably because some of the RS carbon was assimilated (probably via CO_2_) by the plants [20], [21]. However, the difference was only a few permil and did not prevent computation of flux partitioning, since the difference to the δ^13^C of the labeled RS was quite large.

In summary, application of labeled RS may be a convenient technique to determine flux partitioning in rice fields on a routine basis. The determination requires in total three planted field plots and three unplanted ones, i.e., two RS treatments and one untreated control, everything with appropriate replication. Technical installation is not required. Hence, it should be feasible to increase the data basis on the partitioning of CH_4_ production from ROC, RS and SOM on a regional and seasonal scale. This will help improving process-based modeling of CH_4_ emission from rice fields.
